# Inspiratory muscle training for people with spinal cord injury: An implementation study

**DOI:** 10.1177/02692155261418967

**Published:** 2026-03-12

**Authors:** Keira E Tranter, Lisa A Harvey, Lydia W Chen, Lynn Blecher, Jacqui White, Jamie Li, Claire L Boswell-Ruys, Marnie Graco, David J Berlowitz, Joanne V Glinsky

**Affiliations:** 1 The John Walsh Centre for Rehabilitation Research, The University of Sydney, Kolling Institute, NSW, Australia; 2 Spinal Injuries Unit, Royal North Shore Hospital, NSW, Australia; 3 Spinal Injuries Unit, Prince of Wales Hospital, NSW, Australia; 4 Spinal Injuries Unit, Royal Rehab, NSW, Australia; 5 Neuroscience Research Australia, NeuRA, Sydney, NSW, Australia; 6 Department of Physiotherapy, University of Melbourne, Victoria, Australia; 7 Institute for Breathing and Sleep, Austin Health, Victoria, Australia

**Keywords:** Physiotherapy, spinal cord injury, respiratory, inspiratory muscle training, implementation, translational research

## Abstract

**Objective:**

Australian and New Zealand Guidelines for the Physiotherapy Management of People with Spinal Cord Injury recommend the use of inspiratory muscle training for people with spinal cord injury and respiratory muscle weakness. The aim of this study was to evaluate if tailored implementation strategies increased provision of inspiratory muscle training by physiotherapists.

**Design:**

A pre–post implementation study with baseline, post and follow-up measures.

**Setting:**

Three spinal units in Sydney, Australia.

**Participants:**

Twenty-one physiotherapist-participants and 68 patient-participants across three spinal units.

**Intervention:**

Tailored, evidence-based, multi-faceted implementation strategies to improve physiotherapists’ provision of inspiratory muscle training, delivered over a 6-week period (the implementation phase).

**Main measures:**

Physiotherapists’ provision of inspiratory muscle training was measured via a standardised audit tool. Medical records were audited at baseline, immediately after the implementation phase and then two months later to determine whether patients had been provided inspiratory muscle training.

**Results:**

Ninety-four medical records were audited of 68 patient-participants over the study period. Data at baseline indicated that inspiratory muscle training was only provided to 20% of eligible patient-participants. This improved to 91% immediately post the 6-week implementation phase but reduced to 72% two months later.

**Conclusions:**

Tailored, evidence-based implementation strategies increased the provision of inspiratory muscle training by physiotherapists. These strategies can be adapted to different health care settings to improve physiotherapists’ provision of inspiratory muscle training for the respiratory management of people with spinal cord injury.

The study was prospectively registered with the Australian New Zealand Clinical Trials Registry (https://www.anzctr.org.au/. ACTRN: 12623001106628).

## Introduction

There are many physiotherapist-administered treatments to improve respiratory function in people with spinal cord injury, but surprisingly few are supported by evidence. One intervention that is well supported by evidence is inspiratory muscle training. There are numerous randomised controlled trials and a Cochrane review that indicates inspiratory muscle training improves respiratory muscle strength and lung volumes in people with spinal cord injury.^1–11^ More recently, the Australian and New Zealand Guidelines for the physiotherapy management of people with spinal cord injury recommended the use of inspiratory muscle training for people with spinal cord injury and respiratory muscle weakness.^12^ Despite evidence of effectiveness, inspiratory muscle training is not widely used in clinical settings within Australia.

The difficulties with translating evidence into clinical settings are well documented.^13^ Successful implementation of evidence requires identification of the barriers and facilitators (also known as ‘determinants’) to practice. It also requires that these determinants are addressed with targeted implementation strategies.^14^ Importantly, implementation strategies have been found to be most effective when they are multi-faceted and specifically tailored to the needs of an individual setting.^15,16^ For example, they may include the combination of audit feedback, education and the use of a clinical champion. Multi-faceted, implementation strategies are often required to address determinants and facilitate behaviour change at the individual, service and/or organisational level.^17–20^

Although there is mounting evidence to support the efficacy of inspiratory muscle training for improving muscle strength and lung volumes in people with spinal cord injury, it is not part of routine clinical practice in the three spinal units in Sydney, Australia. Therefore, the primary aim of this study was to evaluate if implementation strategies increase provision of inspiratory muscle training by physiotherapists. The secondary aim of this study was to capture physiotherapists’ beliefs about inspiratory muscle training and the implementation process.

## Methods

**Figure 1.**
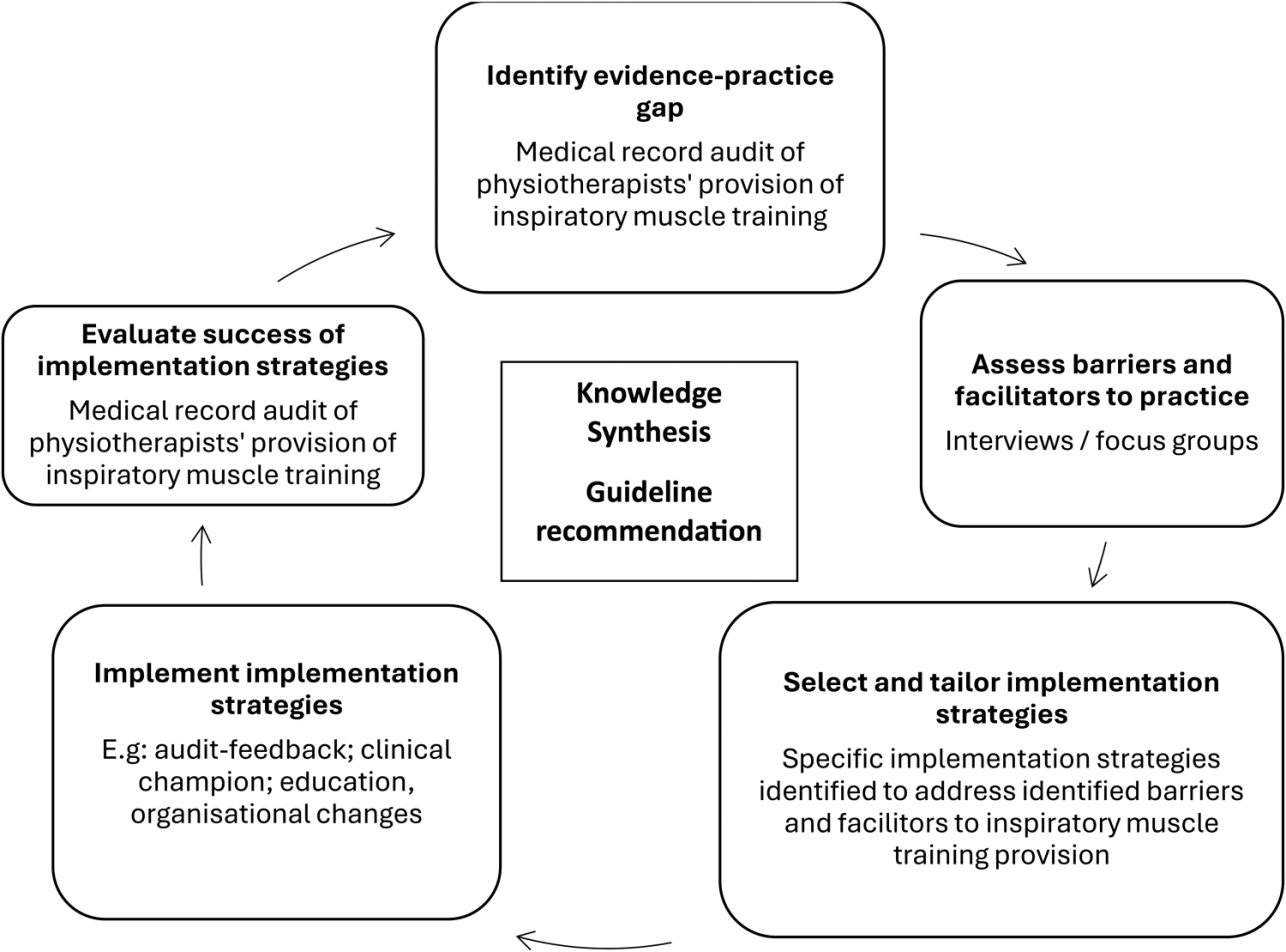
Flow of study using the knowledge to action framework.

A pre–post implementation study with a 2-month follow-up was conducted in the three spinal units in Sydney, Australia (these service the state of New South Wales, with a population of 8.41 million). The study was guided by the Knowledge to Action Framework^21,22^ where the baseline study measures were used to identify the evidence-practice gap (see Figure 1). The study commenced in November 2023 and was completed in July 2024. The study was prospectively registered with the Australian New Zealand Clinical Trials Registry (ACTRN 12623001106628). Ethics approval was gained from the Northern Sydney Local Health District Human Research Ethics Committee (ETH00991) and governance obtained at each site.

Informed consent was obtained from physiotherapists and patients prior to commencement of the study. Physiotherapist-participants were eligible for inclusion if they were providing inpatient services at one of the spinal units. Physiotherapists providing temporary relief for other staff were excluded from the study. Patient-participants were eligible for inclusion if they were over the age of 16; had an injury at T6 neurological level or above (American Spinal Injury Association Impairment Scale A, B, C or D) according to the International Standards of Neurological Classification of Spinal Cord Injury^23^ or T11 and above with comorbidity affecting respiratory function. Patient-participants were excluded if they did not speak sufficient English to provide informed consent or were unable to cooperate (e.g. serious medical condition, cognitive impairment, drug dependency, psychiatric illness or behavioural problems).

Implementation strategies were rolled out over a 6-week period at each of the three spinal units after the baseline data were collected. The details of the strategies and how they were implemented are outlined in Table 1. In brief, the key strategies included audit-feedback, education and use of a clinical champion. Implementation strategies were developed following analysis of qualitative data from semi-structured focus groups and interviews of physiotherapists and patients respectively. Specifically, interviews were developed and analysed using the Theoretical Domains Framework to identify the specific determinants of practice for both physiotherapists and people with spinal cord injury according to 14 factors (domains) that may impact implementation. These data were then mapped to a behaviour change model (COM-B; Capability, Opportunity, Motivation – Behaviour) to identify specific strategies to support implementation of inspiratory muscle training. Strategies were refined and applied to the individual spinal unit in consultation with the physiotherapists. Support was provided by study staff to encourage the use of the implementation strategies and address any concerns raised by the physiotherapists during the 6-week implementation phase. The physiotherapists across the three spinal units agreed to aim for 75% of eligible patient-participants being provided with inspiratory muscle training by the end of the implementation phase.

**Table 1.** Implementation strategies used to promote inspiratory muscle training provision by physiotherapists.

Implementation strategy	Implementation intervention function	Who?	How?	When?
Educational handout	Education	For patients	Provided to the patients by the treating physiotherapist	Initial provision of inspiratory muscle training and referred to as necessary – used to supplement any training provided by the treating physiotherapists
		For physiotherapists	Provided to all physiotherapists	Before the start of implementation phase or when commencing in the spinal unit
Educational session from study staff	Education	For physiotherapists	Online and face to face sessions to each participating spinal unit	Before the start of the implementation phase
Weekly reminder systems	Environmental restructuring	For physiotherapists	At meetings or via email	One single nominated day of the week each week of the implementation phase
Training in the use, prescription and progression of inspiratory muscle training	Training	For physiotherapists	Training from study staff, senior physiotherapists or clinical champion on the use and prescription of inspiratory muscle training as well as the progression	Before the start of the implementation phase and as required for new staff
Templates for handovers and medical record documentation	Enablement	For physiotherapists	Templates created and embedded within existing systems to facilitate documentation	Each time inspiratory muscle training documentation is required in the medical record or for a handover
Documentation guide	Enablement	For physiotherapists	Agreed minimum documentation requirements clearly documented/presented in education material based on training fidelity descriptors	Developed within pre-existing documentation systems
Training to be provided in the gym setting	Enablement, Environmental restructuring	For patients and physiotherapists	Encourage inspiratory muscle training provision in the gym setting	At least four times per week
Clinical Champion	Modelling	Nominated staff member from spinal unit	Model inspiratory muscle training use in gym setting, perform audit and provide feedback to staff, support education and training	Performed the weekly audit-feedback during implementation phase; frequency during the follow-up period at the discretion of the spinal cord injury unit. Education and training was provided as necessary
Change of Business Rule to allow inspiratory muscle training use outside of ward setting	Enablement, Environmental restructuring	For physiotherapists	Support for inspiratory muscle training provision in gym setting required from medical specialists leading to change in workplace policies (Post COVID-19 pandemic)	Before commencement of the implementation phase
Audit-feedback	Environmental restructuring	For physiotherapists	Weekly clinical notes audit of random selection of eligible patients were performed by clinical champion	The results of the audit-feedback strategy would be fed back to the team to increase awareness of practice to encourage increased uptake of inspiratory muscle training
Weekly review of list of inpatients	Environmental restructuring	Physiotherapist/Clinical Champion	Screen for eligible patients to receive inspiratory muscle training	The individual spinal unit nominated a day of the week where they reviewed their patient list
Visual Posters/Whiteboards	Environmental restructuring	For patients and physiotherapists	Visual reminders for physiotherapists of who has or who requires inspiratory muscle training; visual prompts for patients to remind them to do inspiratory muscle training	These were set-up in in gym and/or office settings depending on what worked best for the individual spinal unit.
Guideline for provision of inspiratory muscle training	Restriction	For physiotherapists	The prescribing criteria were developed in consultation with all spinal units and study staff. The guideline for inspiratory muscle training provision was agreed upon by physiotherapists from each spinal unit	Person with spinal cord injury: T6 or above of any severity; or T11 and above with respiratory comorbidity. 3–5 × 10–12 breaths, daily, at least 4 times per week, at a modified BORG ≥ 4; progressed at least weekly to maintain modified BORG ≥ 4

Outcomes were taken at three time points: baseline; immediately after the implementation phase (post implementation); and 2-months from the completion of the implementation phase (follow-up). Physiotherapist satisfaction with the implementation process was only measured at follow-up.

The primary outcome was the percentage of eligible patient-participants who were provided inspiratory muscle training by their physiotherapist. This was measured by medical record audits capturing if inspiratory muscle training had been provided in the two weeks prior to the audit. Provision was judged as ‘yes’ or ‘no’ and converted to a percentage. Descriptive data about the provision of inspiratory muscle training, i.e., training fidelity as per current evidence (intensity, dosage and progression) were also extracted.^2^

There were four secondary outcomes. All secondary outcome measures were completed by physiotherapist-participants and captured via online survey (REDCap v13.0).

First, physiotherapist-participants were asked to rate the Acceptability, Appropriateness and Feasibility of Inspiratory Muscle Training using a 5-point Likert scale (1–5), with one meaning ‘completely disagree’ and five meaning ‘completely agree’. This measure has been validated and shown to be reliable with good responsiveness to change.^24^ This consisted of three parts which the physiotherapist-participants were required to rate if they believed that inspiratory muscle training was acceptable, appropriate and feasible to implement. Scores were summed and averaged with a higher score indicating a more positive belief about inspiratory muscle training.

Second, physiotherapist-participants’ knowledge of the recommendation made in the Australian and New Zealand Clinical Practice Guidelines for the physiotherapy management of people with spinal cord injury was captured. To do this physiotherapist-participants were asked to correctly identify the strength and direction of the guideline recommendation (i.e., strong for, weak for, strong against, weak against).

Third, physiotherapist-participants were asked to complete four sections of the NoMAD Survey,^25^ which is an implementation measure based on Normalisation Process Theory. This was used to capture physiotherapist-participants perspectives on inspiratory muscle training and if these perspectives changed because of the implementation process.

Lastly, physiotherapist-participants satisfaction with the guideline implementation process was self-rated on an 11-point visual analogue scale (0–10), with zero meaning ‘*not at all satisfied’* and 10 meaning ‘*very satisfied’*. The staff also had the opportunity to provide feedback on the implementation process.

All data were analysed descriptively using Excel. Nominal data are expressed in percentages, and continuous data are expressed as mean (and SDs).

## Results

Twenty-one physiotherapists participated in the study across three spinal units. Eleven physiotherapists remained on the spinal units for the duration of the study and the other physiotherapists moved on and off the units at varying times. The median (interquartile range, IQR) experience in the field of spinal cord injury was 3 years (2 to 11). Characteristics of the 11 physiotherapist-participants whose practices were audited at all three time points (baseline, post and follow-up) can be found in Table 2. Characteristics of all physiotherapist-participants at each time point (baseline, post implementation and follow-up) can be found in Supplemental File 1.

**Table 2.** Characteristics of physiotherapist-participants.

	Physiotherapist-participants (*n* = 11)
Age (years), median (IQR)	34 (29 to 37)
Gender, Female: Male	10:1
Experience as a physiotherapist (years), median (IQR)	9 (5 to 15)
Experience working as a physiotherapist with people with spinal cord injury (years), median (IQR)	3 (2 to 11)
Oversees the provision of inspiratory muscle training (management role within the spinal unit), *n* (%)	7 (64)
Provides inspiratory muscle training to people with spinal cord injury, *n* (%)	10 (91)

IQR: interquartile range; *n*: number of physiotherapist-participants.

Ninety-four medical record audits of 68 patient-participants were conducted over the study period (see Table 3). Patient-participants median age and time since injury were 61 (34 to 69) years and 3 (2 to 4) months. Patient-participants had American Spinal Injury Association Impairment Scale A (*n* = 21, 31%), B (*n* = 10, 15%), C (*n* = 14, 21%) or D (*n* = 23, 34%) lesions with neurological levels from C1 to T8 (see Table 3).

**Table 3.** Characteristics of patient-participants audited at each time point and overall.

	Guideline audit time point			
	Baseline (*n* = 30)	Post implementation (*n* = 35)	Follow-up (*n* = 29)	Overall** (*n* = 68)
Age (years), median (IQR)	61 (37 to 71)	60 (31 to 67)	62 (37 to 69)	61 (34 to 69)
Gender, male: female (male, %)	27:3 (90)	32:3 (91)	25:4 (86)	61:7 (90)
Time since injury (months), median (IQR)	3 (2 to 7)	3 (2 to 5)	4 (2 to 6)	3 (2 to 4)
Neurological level, *n* (%)*				
C1–C4	19 (63)	10 (29)	13 (45)	30 (44)
C5–C8	4 (13)	9 (26)	9 (31)	15 (22)
T1–T6	6 (20)	14 (40)	5 (17)	20 (29)
T7–T11	1 (3)	2 (6)	2 (7)	3 (4)
American Spinal Injury Association Impairment Scale classification, *n* (%)*				
A	8 (27)	13 (37)	9 (31)	21 (31)
B	7 (23)	5 (14)	5 (17)	10 (15)
C	7 (23)	6 (17)	4 (14)	14 (21)
D	8 (27)	11 (31)	11 (38)	23 (34)

IQR: interquartile range; *n*: number of physiotherapist-participants.

*Percentage sums may not add exactly to 100 due to the effects of rounding.

**NB: overall of *n* is less than the addition of each time point because some patient-participants were audited over multiple time-points.

An evidence to practice gap was identified from the baseline medical record audits, with only 20% of eligible patient-participants receiving inspiratory muscle training. When inspiratory muscle training was provided, it was only prescribed at the appropriate intensity and dosage 17% of the time (Training fidelity: modified BORG ≥4 or Maximal Inspiratory Pressure of ≥40%; dosage of 3 × 10–12 breaths daily at least four times per week with appropriate progression).

Immediately following the 6-week implementation period, physiotherapists’ provision of inspiratory muscle training had improved to 91%. When inspiratory muscle training was provided, it was prescribed with an appropriate training fidelity more than 94% of the time. Two months following the cessation of the implementation phase, physiotherapists’ provision of inspiratory muscle training had reduced to 72% and it was prescribed with an appropriate training fidelity 76% of the time (see Table 4).

**Table 4.** Physiotherapists’ adherence to the guideline recommendation (primary outcome).

	Baseline (*n* = 30)	Post implementation (*n* = 35)	Follow-up (*n* = 29)
Number (percentage) of eligible patient-participants who received inspiratory muscle training, *n* (%)	6 (20)	32 (91)	21 (72)

This was captured by the number (percentage) of eligible patient-participants prescribed inspiratory muscle training by their treating physiotherapist at baseline, post implementation and follow-up.

*n*: number of patient-participants.

**Figure 2.**
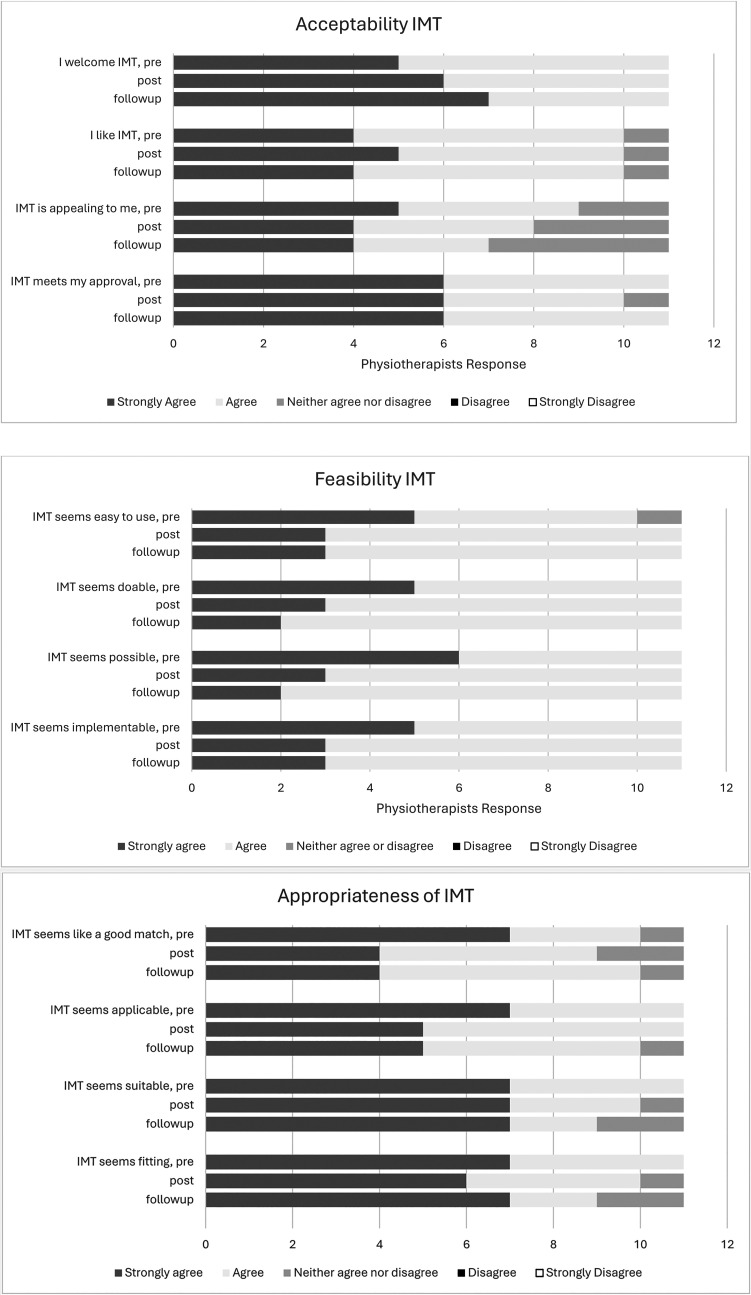
Measures of acceptability, appropriateness and feasibility of inspiratory muscle training (IMT) at baseline, post implementation and at follow-up.

Secondary outcome measures of Acceptability, Appropriateness and Feasibility demonstrated physiotherapists’ beliefs that inspiratory muscle training is an acceptable and appropriate intervention as well as feasible to implement (see Table 5 and Figure 2). These beliefs did not change over time (see Supplemental File 2). Furthermore, these beliefs were reinforced by physiotherapists’ responses to portions of the NoMAD survey, whereby inspiratory muscle training was deemed by the physiotherapists as a familiar intervention and accepted as part of usual care (see Table 6). The NoMAD survey also demonstrated that physiotherapists had a greater agreement following the implementation phase for key areas that enabled inspiratory muscle training use, such as management support, resources, training, and confidence in others ability to perform inspiratory muscle training (see Supplemental File 3).

**Table 5.** Secondary outcomes at baseline, post implementation and follow-up.

Outcome	Baseline (*n* = 11)	Post implementation (*n* = 11)	Follow-up (*n* = 11)
Acceptability of Intervention Measure (1–5 points): mean, (SD)	4 (1)	4 (1)	4 (1)
Intervention Appropriateness Measure (1–5 points): mean (SD)	5 (1)	4 (1)	4 (1)
Feasibility of Intervention Measure (1–5 points): mean (SD)	4 (1)	4 (0)	4 (0)
Number of physiotherapists who knew The Guideline recommendation for inspiratory muscle training, *n* (%)	6 (55)	10 (91)	10 (91)

*n:* number of physiotherapist-participants surveyed; SD: standard deviation.

**Table 6.** Summary of results for physiotherapist-participants’ responses from the NoMAD (part B) survey for inspiratory muscle training (baseline, post implementation and follow-up).

	Baseline (*n* = 11)	Post implementation (*n* = 11)	Follow-up (*n* = 11)
Familiarity of intervention (0–10 points), mean (SD)	8 (1)	8 (1)	9 (1)
Intervention part of normal clinical practice (0–10 points), mean (SD)	8 (2)	7 (2)	8 (2)
Intervention will become part of clinical practice (0–10 points), mean (SD)	9 (2)	8 (2)	8 (1)

*n:* number of surveys completed by physiotherapist-participants. SD: standard deviation.

Knowledge of the recommendation for inspiratory muscle training within the Australian and New Zealand Clinical Practice Guideline improved from 55% at baseline, to 91% immediately following the implementation phase which was maintained at the 2-month follow-up. Overall, the physiotherapists were satisfied with the implementation process with a mean (SD) satisfaction of 8 (1) out of 10.

## Discussion

This study demonstrated that tailored, evidence-based, multi-faceted implementation strategies are effective at increasing physiotherapists’ provision of inspiratory muscle training. Consequently, facilitating the uptake of the Australian and New Zealand Clinical Practice Guideline recommendation into clinical practice. Following the 6-week implementation phase, there were substantial improvements in the provision of inspiratory muscle training to eligible patient-participants from baseline, with rates of provision increasing from 20% to 91%. This exceeded the target goal of 75% set by all three spinal units. Following the 2-month follow-up period where no input from the study team were provided, physiotherapists’ adherence to inspiratory muscle training provision decreased to 72% demonstrating implementation decay.

The success of the study in improving the provision of inspiratory muscle training by physiotherapists is likely due to three key implementation strategies (audit-feedback, education and use of a clinical champion). Audit-feedback is known to be a successful implementation strategy when the delivery of a particular intervention is low.^26^ In our study audit-feedback (written and verbal) was provided weekly to all physiotherapists during the implementation phase. Study staff supported the initial audit-feedback loops which were then taken over by the clinical champion at each site. As such, the feedback was provided by a peer, in a positive and supportive manner, which is likely to have encouraged the physiotherapists to change their behaviour and provide inspiratory muscle training more regularly. The second key strategy, education, focused on improving physiotherapists’ knowledge of inspiratory muscle training. It was provided in two ways, printed educational resources and an educational session provided by study staff.^27,28^ The educational resources provided a summary of the evidence, key prescribing criteria for the provision of inspiratory muscle training as well as how to set and progress the training intensity. Education provided by study staff ensured physiotherapists were confident using the training devices and addressed any gaps in skill as necessary. Lastly, the use of a clinical champion was important to model positive behaviour in the clinical setting.^29^ The clinical champion was responsible for promoting accountability within the physiotherapy team by reminding staff each week to ensure all eligible patients received inspiratory muscle training. In addition, setting a target goal of 75% adherence may have also provided motivation for physiotherapists.

Adherence with suggested implementation strategies throughout the study period was not measured, so the reasons for implementation decay can only be hypothesised. This was most likely due to the physiotherapists’ reduced sense of accountability. Physiotherapists reported that the audit-feedback loop was useful to provide objective feedback on current clinical practice during the implementation phase. However, during the 2-month follow-up period, the frequency of audit-feedback was left to the discretion of the spinal units. The spinal units that continued with audit-feedback tended to continue providing inspiratory muscle training over the follow-up period. This is reinforced by evidence supporting the regular use of audit-feedback.^26,30,31^

This study is not without limitations. We only provided the implementation strategies over a 6-week period. This may not have been sufficient to sustain the change in physiotherapists’ behaviour. Despite this, a substantial proportion of physiotherapists continued to provide inspiratory muscle training to eligible patients as reflected in the 72% adherence at follow-up. A second round of interviews with physiotherapist-participants after the follow-up period may have been valuable in providing insight into barriers to sustainment of behaviour change and challenges faced by physiotherapists. This could be used to inform ongoing implementation strategies. Further research could also determine the effectiveness of longer implementation periods and ongoing periodic follow-up on physiotherapists’ behaviour. Furthermore, the study would have been strengthened by the inclusion of more sites, physiotherapists and patients. Nonetheless, this study demonstrates that tailored implementation strategies can increase physiotherapists’ provision of inspiratory muscle training in clinical practice. It also highlights the need for implementation studies to facilitate translation of evidence into practice.

Despite evidence supporting the effectiveness of inspiratory muscle training for people with spinal cord injury, and physiotherapists’ beliefs of inspiratory muscle training being an acceptable, appropriate and feasible intervention to implement, it cannot be assumed that it is being provided as part of physiotherapists’ routine practice. This study demonstrates the need for tailored strategies to support change in physiotherapists’ behaviour to promote the provision of inspiratory muscle training. The strategies used within this study were effective for increasing physiotherapists’ provision of inspiratory muscle training and can be tailored to individual healthcare contexts to promote the use inspiratory muscle training to optimise the management of people with spinal cord injury.

**Clinical messages**

- The implementation of inspiratory muscle training into clinical practice is best supported by tailored, evidence-based, multi-faceted strategies.
- Implementation strategies used in this study can be adapted to other health contexts to promote physiotherapists’ provision of inspiratory muscle training.

## Supplemental Material

sj-docx-1-cre-10.1177_02692155261418967 - Supplemental material for Inspiratory muscle training for people with spinal cord injury: An implementation study Supplemental material, sj-docx-1-cre-10.1177_02692155261418967 for Inspiratory muscle training for people with spinal cord injury: An implementation study by Keira E Tranter, Lisa A Harvey, Lydia W Chen, Lynn Blecher, Jacqui White, Jamie Li, Claire L Boswell-Ruys, Marnie Graco, David J Berlowitz and Joanne V Glinsky in Clinical Rehabilitation
