# Supplementary material for: Inspiratory muscle training for people with spinal cord injury: An implementation study
Source: Clin Rehabil. 2026 Mar 12;40(7):972–84. doi: 10.1177/02692155261418967 (PMC13283492; doi:10.1177/02692155261418967)

Supplementary File 1: Characteristics of Physiotherapist-participants at baseline, post-implementation and follow-up. n= number of physiotherapist-participants surveyed

|  | Baseline  (n=21) | Post-implementation  (n=12) | Follow-up  (n=12) |
| --- | --- | --- | --- |
| Age (years), median (IQR) | 31 (26 to 36) | 33 (26 to 36) | 33 (26 to 36) |
| Sex (Female:Male) | 20:1 | 11:1 | 11:1 |
| Experience as a physiotherapist (years), median (IQR) | 5 (2 to 10) | 7 (3 to 14) | 7 (2 to 14) |
| Experience working as a physiotherapist with people with SCI (years), median (IQR) | 2 (1 to 9) | 3 (1 to 11) | 3 (1 to 11) |
| Involved in managing inspiratory muscle training, n (%) | 12 (57) | 7 (58) | 7 (58) |
| Involved in delivering inspiratory muscle training, n (%) | 20 (95) | 10 (83) | 11 (92) |

IQR: Interquartile Range

Supplementary File 2: Secondary outcomes at baseline, post implementation and follow-up; n= number of physiotherapist-participants surveyed

| **Outcome** | **Baseline**  **(n=21)** | **Post implementation**  **(n= 12)** | **Follow-up**  **(n=12)** |
| --- | --- | --- | --- |
| Acceptability of Intervention Measure, mean (SD) 1-5 | 4 (0) | 4 (1) | 4 (1) |
| Intervention Appropriateness Measure, mean (SD) 1-5 | 4 (1) | 4 (1) | 4 (1) |
| Feasibility of Intervention Measure, mean (SD) 1-5 | 4(1) | 4 (0) | 4 (0) |
| Number of physiotherapists who knew the Guideline recommendation for inspiratory muscle training, n (%) | 10 (48) | 11 (92) | 11 (92) |

SD: Standard deviation

Supplementary File 3: Physiotherapists’ responses to the NOMAD survey baseline, post and follow-up implementation phase


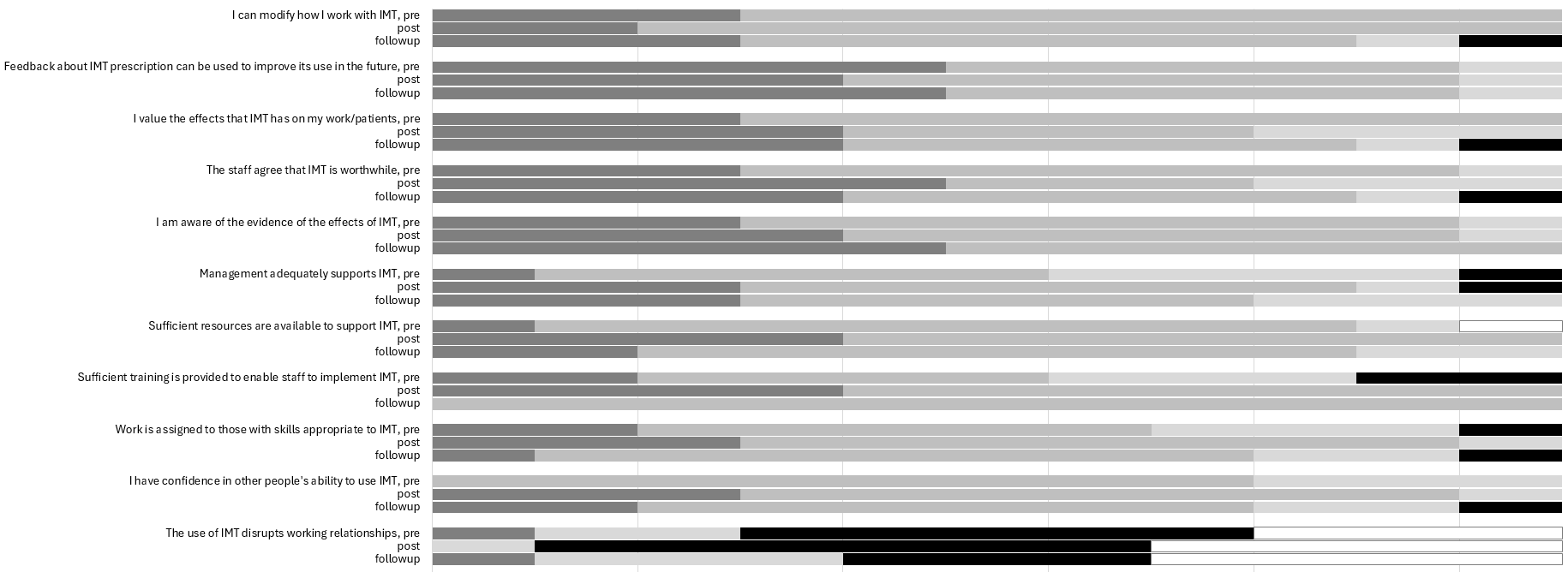


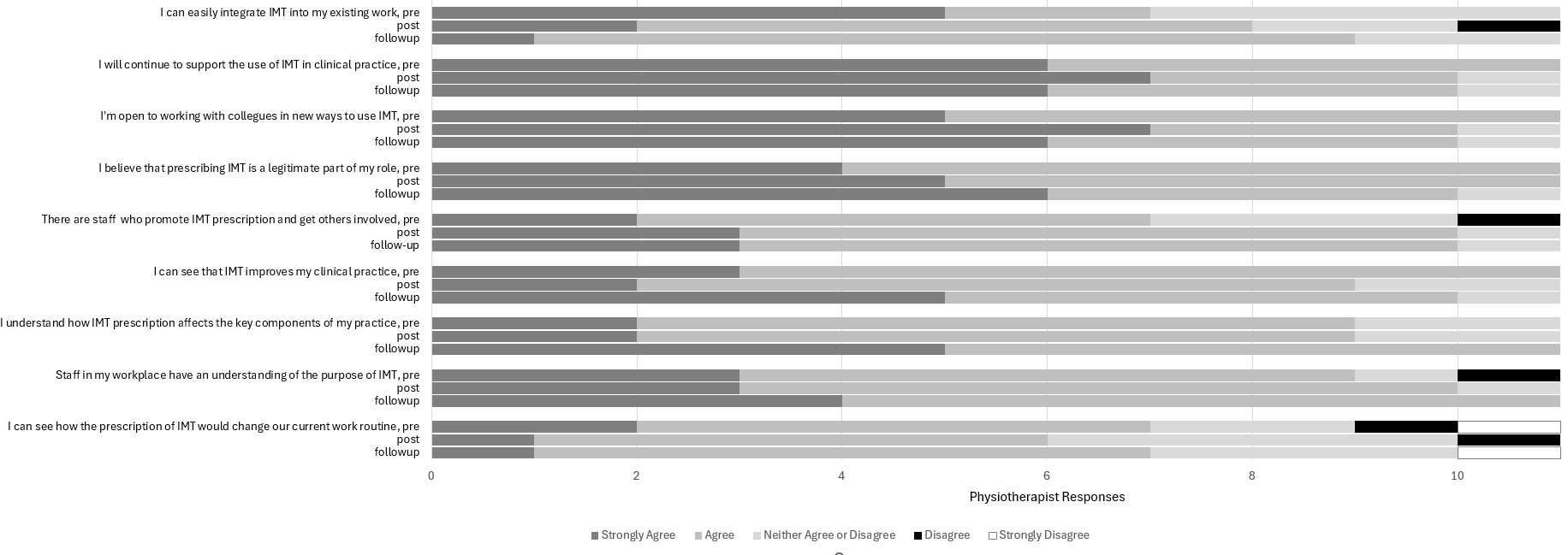


## Supplementary File 4: Education Handout for Physiotherapist-participants


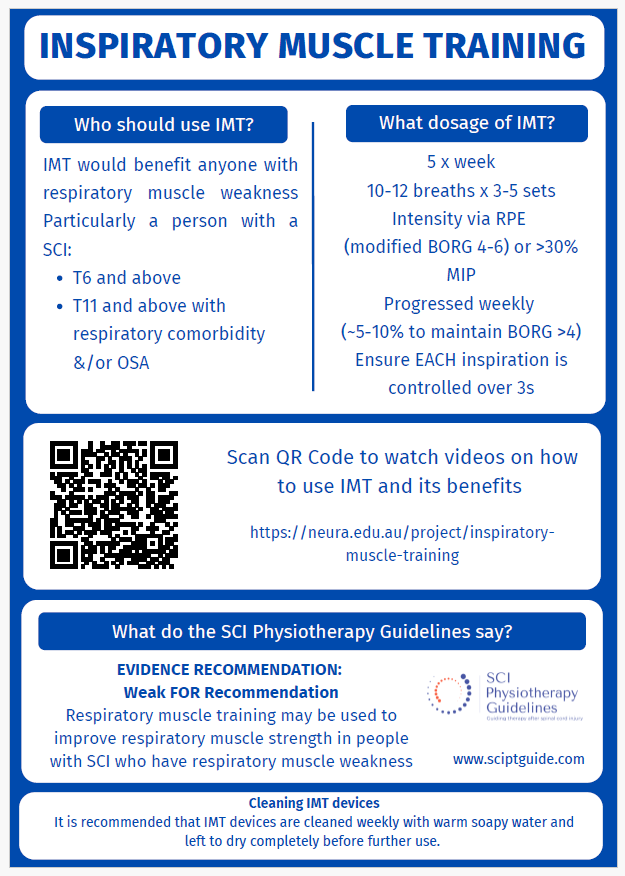


## Supplementary File 5: Education Handout for Consumer-participants


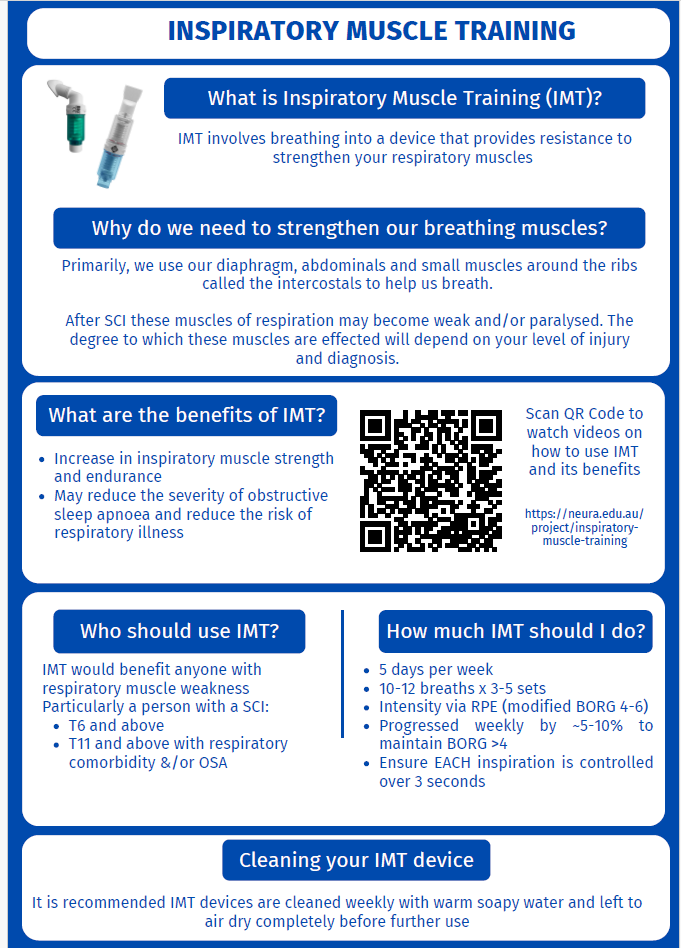

Supplement: sj-docx-1-cre-10.1177_02692155261418967 - Supplemental material for Inspiratory muscle training for people with spinal cord injury: An implementation study [file sj-docx-1-cre-10.1177_02692155261418967.docx]
